# Enhancing acute flaccid paralysis surveillance system towards polio eradication: reverse cold chain monitoring in Nigeria, 2017 to 2019

**DOI:** 10.11604/pamj.supp.2021.40.1.27534

**Published:** 2021-11-12

**Authors:** Samuel Luka Abbott, Abdullahi Walla Hamisu, Saheed Gidado, Sume Gerald Etapelong, Aboyowa Arayuwa Edukugho, Isiaka Ayodeji Hassan, Kabir Yusuf Mawashi, David Nadeba Bukbuk, Marycelin Baba, Adeniji Johnson Adekunle, Usman Saidu Adamu, Eunice Damisa, Ndadilnasiya Endie Waziri, Wiedad Roodly Archer, Richard Franka, Eric Wiesen, Fiona Braka, Omotayo Bolu, Richard Banda, Faisal Shuaib

**Affiliations:** 1 African Field Epidemiology Network, Abuja, Nigeria,; 2 World Health Organization, Abuja, Nigeria,; 3 National Primary Health Care Development Agency, Abuja, Nigeria,; 4 Polio Laboratory, University of Maiduguri, Borno State, Nigeria,; 5 Polio Laboratory, University College Hospital Ibadan, Oyo State, Nigeria,; 6 Centre for Disease Control and Prevention, Atlanta, Georgia,; 7 Centre for Disease Control and Prevention, Nigeria Office, Abuja, Nigeria

**Keywords:** AFP surveillance, reverse cold chain, enterovirus, poliovirus, temperature excursion

## Abstract

**Introduction:**

Highly sensitive acute flaccid paralysis (AFP) surveillance is critical for detection of poliovirus circulation and documentation for polio-free certification. The reverse cold chain (RCC) is a system designed to maintain stool specimens in appropriate temperature for effective detection of poliovirus in the laboratory. We monitored the RCC of AFP surveillance in Nigeria to determine its effectiveness in maintaining viability of enterovirus.

**Methods:**

A descriptive cross-sectional study was conducted from November 2017 to December 2019. We included AFP cases from 151 Local Government Areas and monitored RCC of paired stool specimens from collection to arrival at laboratories. The national guideline recommends RCC temperature of +2 to +8°C and a non-polio enterovirus (NPENT) detection rate of ≥10%. We analyzed data with Epi Info 7, and presented results as frequencies and proportions, using Chi-square statistic to test for difference in enterovirus isolation.

**Results:**

Of the 1,042 tracked paired stool specimens, 1,038(99.6%) arrived at the laboratory within 72 hours of collection of second specimen, 824(79.1%) were maintained within recommended temperature range, and 271(26%) yielded enteroviruses: 200(73.8%) NPENT, 66(24.4%) Sabin, 3(1.1%) vaccine derived poliovirus type 2 and 2(0.7%) mixture of Sabin and NPENT. The NPENT and Sabin rates were 19.2% and 6.7% respectively. Twenty-five percent of 824 specimens maintained within recommended temperature range, compared with 29.8% of 218 specimens with temperature excursion yielded enteroviruses (P=0.175).

**Conclusion:**

the RCC of AFP surveillance system in the study area was optimal and effective in maintaining the viability of enteroviruses. It was unlikely that poliovirus transmission was missed during the intervention.

## Introduction

Poliomyelitis is a viral disease targeted for eradication. It is caused by the poliovirus which belongs to the genus enteroviruses. The global effort for the eradication of wild type poliovirus reduced the number of polio endemic countries from 125 in 1988 to 3 (Pakistan, Afghanistan, and Nigeria) in 2016 [1,2]. The strategy for achieving polio eradication and maintaining polio-free status has two essential components: achieving and maintaining high population immunity through vaccination with polio vaccines, and detecting and responding to poliovirus through a highly sensitive surveillance system [3]. The Global Certification Commission (GCC) established a set of criteria that defines poliovirus certification standards; quality acute flaccid paralysis (AFP) surveillance is a key component of these criteria. Highly sensitive AFP surveillance, including immediate AFP case investigation and stool specimen collection, are critical for the detection of poliovirus. It is also critical for documenting the absence of poliovirus circulation for polio-free certification [4]. The two main indicators for assessing AFP surveillance sensitivity are the annualized non-polio AFP (NPAFP) rate per 100,000 children under 15 years per year and percentage stool adequacy. Adequate stool is two stool specimens collected 24-48 hours apart, within 14 days after onset of paralysis and arriving the laboratory in good condition. Good condition means that upon arrival there are frozen icepacks or a temperature indicator showing < 8°C in the container, each sample volume is adequate (>8 grams), there is no evidence of leakage or desiccation and there is proper documentation. The World Health Organization (WHO)´s African Region certification standards for these indicators are NPAFP rate of ≥2/100,000 and stool adequacy of ≥80% [4].

Nigeria achieved zero wild poliovirus (WPV) case for 23 months since the last case was reported on 24 July 2014, and was subsequently delisted from polio endemic countries on 15 September 2015 by the WHO [5,6]. However, between July and August 2016, four wild poliovirus type 1 (WPV1) cases were isolated in Borno State, northeast Nigeria, with genetic sequencing indicating prolonged undetected transmission of the viruses - a consequence of low population immunity and AFP surveillance gaps due to the insurgency related insecurity in the northeast region of Nigeria which has negatively affected healthcare delivery system in general and public health surveillance in particular [5]. An AFP surveillance review conducted as part of the outbreak response recommended strengthening of the surveillance system and in particular to monitor the Reverse Cold Chain (RCC) of the AFP surveillance system.

RCC is a system of storing and transporting AFP stool specimens within the recommended temperature range from the point of collection to arrival at the laboratory [7]. This ensures specimens are stored and transported for optimal viral isolation in the laboratory. To ensure validity of the laboratory testing, stool specimens from suspected AFP cases should be transported to a WHO-accredited laboratory at a temperature range of +2 to +8°C within 72 hours of collection of the second stool specimen [8]. In Nigeria, there are two WHO-accredited polio laboratories located in Ibadan and Maiduguri in southern and northern parts of the country, respectively.

Between November 2017 and December 2019, the Nigeria polio program monitored RCC of AFP stool specimens from point of collection (i.e., health facilities or homes of caregivers) to arrival at the polio laboratories to assess effectiveness in maintaining the viability of enterovirus for isolation, identify and address possible gaps to improve RCC quality, and ensure that poliovirus transmission is not missed. This paper describes this intervention, presents the findings, and articulates the lessons learnt for programmatic improvement.

## Methods

**Figure 1:**
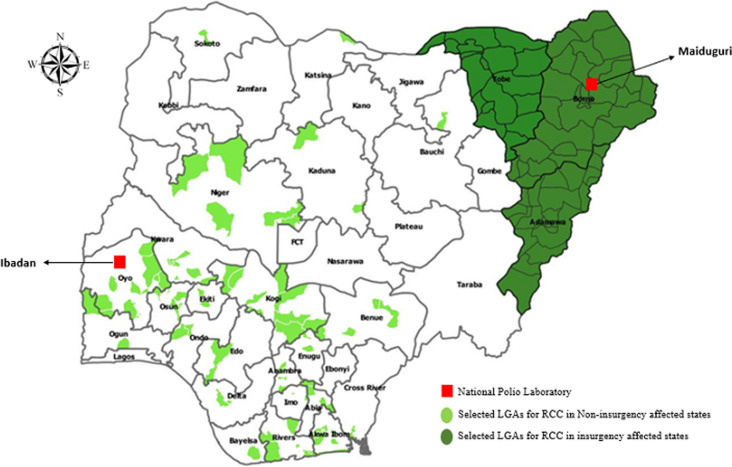
local government areas selected for reverse cold chain monitoring and locations of polio laboratories, Nigeria, November 2017 - December 2019

Study design and area: we conducted a descriptive cross-sectional study between November 2017 and December 2019. The study was conducted in 151 (19.5%) out of the 774 Local Government Areas (LGAs) selected across 26 (72.2%) out of 36 states in Nigeria, and the Federal Capital Territory (FCT) (Figure 1).

Specimen selection and sampling technique: a purposive sampling technique was used to select the 151 LGAs. We conducted a desk review in September 2017 and selected 87 LGAs, including one LGA from Adamawa State, that detected at least an AFP case between epidemiologic week 1 and 35 of 2017 but had zero enterovirus detection rate. Beginning February 2018, all 27 LGAs in Borno and all 17 LGAs in Yobe states, and the remaining 20 LGAs in Adamawa State, were included as recommended by the second outbreak response assessment (OBRA) because Adamawa, Borno and Yobe states are insurgency affected states. Each LGA was assigned only one LogTag to track temperatures of stool specimens from the point of collection to the laboratory. We tracked stool specimens from AFP cases as they were being detected subject to availability of LogTag. In situations where multiple AFP cases were detected in the same LGA at same time, the stool specimens from first detected case was tracked. AFP cases detected when LogTag was already in use to track other specimens or was faulty could not be tracked. According to the guidelines, AFP stool specimens were expected to be transported to the laboratories within 72 hours from the time of collection of the second stool specimen [8] hence the reason for transporting specimen immediately to the laboratory irrespective of the availability of LogTag.

Specimen collection and packaging: two stool specimens weighing approximately eight grams each were collected and placed in a clean container with a screw-on cap to tightly seal the container. The container was labelled with the name, unique identification number of the case, number of specimens (1 and 2), time and date of collection using a water-resistant pen. The stool specimens were placed in a plastic bag and sealed. The completed AFP case investigation form was placed in a separate plastic bag and placed in the stool specimen carrier containing icepacks or carried along by the transporter in a folder.

We used the LogTag® UTRIX-16 multi-use USB temperature recorder (LogTag) [9] to record the temperature of the tracked AFP stool specimens from point of collection to arrival at the laboratory. The LogTag were configured using the LogTag® Analyzer software to record temperature every five minutes over a 30-day period. The lower and upper temperature alarm limits were set at +2 and +8°C, respectively. The LogTags were turned on and placed inside the stool specimen carriers (Giostyle) containing 4 - 8 icepacks before the first specimen was collected and left with the caregiver after detailed handling instructions were provided. Icepacks were replaced every 24 hours until the second stool specimen was collected. The specimens were transported immediately to the state surveillance office and stored in dedicated refrigerators at +2 to +8°C until shipment was arranged. Stool specimens were shipped in carriers with icepacks to the polio laboratories. The basic triple packaging system was used to ship the stool specimens to the laboratories [10]. When the LogTags were separated from the stool specimens, the LogTags temperature readings from the point of collection to arrival at the laboratories were downloaded into a database in the laboratory and returned to the LGA. At the laboratory, the stool specimens were received and stored according to Global Polio Laboratory Network (GPLN) guidelines; virologic analysis were conducted as described in polio laboratory manual [11] and results were updated in the laboratories and AFP databases. The title of the stool specimen couriers was verified and recorded. We defined temperature excursion as temperature greater than +8°C.

Data collection: we collected data using four instruments. We used the standard WHO AFRO AFP case investigation form to collect AFP case related data. The LogTag autogenerated a PDF data output which captured records of temperature readings at regular time intervals including the first and last temperature readings, the highest and lowest temperature readings, as well as the duration of temperature excursion for specimens with temperature readings below and above recommended RCC temperature range. Furthermore, a Microsoft Office Excel 2010 tool was used in the laboratory to summarize weekly data of tracked paired stool specimens. These data elements included state, LGA, transporter of stool specimens, date, and time LogTag was turned on, date and time specimens were collected, transported, and arrived in the laboratories, as well as temperature upon arrival at the laboratories. In addition, we used a semi-structured questionnaire to investigate specimens with RCC temperatures above +8°C at any point between collection and arrival at the laboratory. The semi-structured tool had questions on the condition of the specimen carrier used, the numbers and condition of icepacks used for collection and transportation of specimens, stool specimen collection, packaging, storage, and time of transportation to the polio laboratory. Specimens with temperature excursions were investigated by state surveillance officers to determine the possible cause to guide recommended interventions.

Data management: the weekly summary data (i.e., Microsoft Office Excel 2010 tool) and the PDF readings from the LogTag were cleaned, collated, and shared weekly by the laboratories with the National Surveillance Officer who then analyzed and shared with state surveillance officers who then shared with the LGA surveillance officers. The AFP case based and laboratory information for selected LGAs were obtained from the standard AFP and laboratory databases. Data from the semi-structured questionnaire was used to create an Excel database. We conducted univariate, bivariate, and stratified analysis and results were presented as frequencies and proportions. The Chi Square test was used to compare enterovirus detection in specimens with temperature excursions and those without temperature excursions using Epi Info 7. Values of P<0.05 were considered statistically significant.

**Ethical consideration:** this intervention was conducted as an operational strategy by the Nigerian Polio programme as part of the overall efforts to improve the quality of AFP surveillance to achieve the goal of polio eradication in the country therefore it was classified as non-research.

## Results

**Figure 2:**
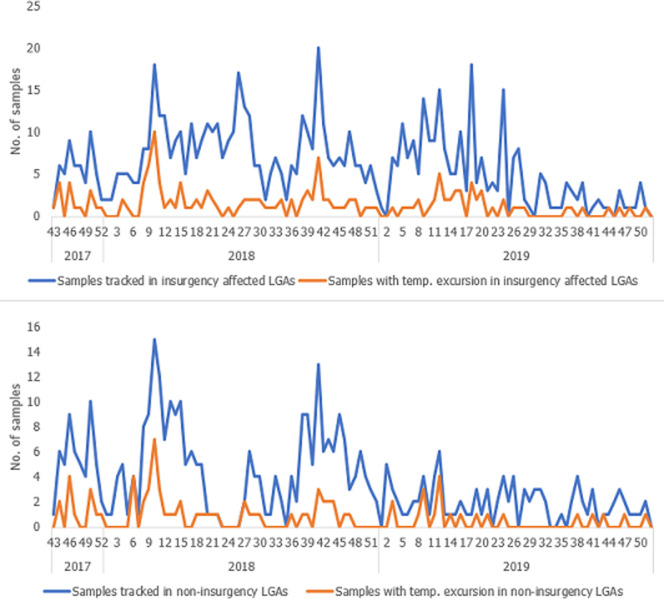
Weekly trend of paired stool specimens tracked, reverse cold chain monitoring, Nigeria, November 2017 to December 2019

A total of 2,315 AFP cases were reported from the 151 LGAs within the study period, out of which paired stool specimens from 1,042(45.0%) AFP cases were tracked from the point of collection to arrival at the polio laboratories and included in analysis (Table 1). All paired stool specimens from the 1,042 AFP cases were collected within 14 days of onset of paralysis, 24 - 48 hours apart. Of the 1,042 AFP paired stool specimens tracked, 440(42.2%) and 602(57.8%) were transported to Ibadan and Maiduguri laboratories, respectively. Of these 1,042 paired stool specimens, 56(5.4%) were tracked from November to December 2017, 652(62.6%) in 2018 and 334(32.0%) in 2019. Overall, 218(21.0%) paired stool specimens had temperature excursions: 10(5.0%) during specimen collection, 168(77.0%) during specimen transportation, and 40(18.0%) during both specimen collection and transportation to the laboratory. Twenty-seven percent (15/56), 21.0% (138/652) and 19.0% (65/334) of tracked paired stool specimens had temperature excursion in 2017, 2018 and 2019, respectively (Figure 2). In 65 LGAs from the three insurgency affected states, 482(79.5%) of 606 tracked paired stool specimens arrived at the laboratory within the recommended temperature range. Overall, these LGAs accounted for 124(56.9%) of the 218 paired stool specimens with temperature excursion, and 482(58.5%) of 824 paired specimens without temperature excursion.

**Table 1:** distribution of tracked paired stool specimens, reverse cold chain monitoring, Nigeria, November 2017 - December 2019

Year	Total paired stool specimens investigated	Paired specimens tracked, n(%)	Ibadan Laboratory		Maiduguri Laboratory	
			Paired specimens tracked n(%)	Tracked paired specimens with temperature excursions n(%)	Paired specimens tracked n(%)	Tracked paired specimens with temperature excursions n(%)
November to December 2017	56	56(100)	47(83.9)	13(27.7)	9(16.1)	2(22.2)
January to December 2018	1214	652(53.7)	264(40.5)	82(31.1)	388(59.5)	56(14.4)
January to December 2019	1045	334(32.0)	129(38.6)	54(41.9)	205(61.4)	11(5.4)
**Total**	**2315**	**1042(45)**	**440(42.2)**	**149(33.9)**	**602(57.8)**	**69(11.5)**

### Transportation of specimens to the laboratories

Of the 1,042 tracked paired stool specimens, 1,038(99.6%) were transported and arrived at the national polio laboratories within 72 hours of collection of the second stool specimen. Six hundred and fifteen (59.0%) of the paired specimens were transported to the national polio laboratories by Disease Surveillance and Notification Officers (DSNOs), 407(39.0%) by Assistant Disease Surveillance and Notification Officers (ADSNOs) and 20(2.0%) by Health Facility Surveillance Focal Person.

### Laboratory analysis of specimens

Of the 1,042 tracked paired stool specimens, 271(26.0%) yielded enterovirus isolates. Of the 271 enterovirus isolates, 200(73.8%) were Non-Polio Enterovirus (NPENT), 66(24.4%) were Sabin, 3(1.1%) were Vaccine Derived Poliovirus type 2 (VDPV2), 2(0.7%) were NPENT and Sabin mixture (Table 2). None of the tracked specimens yielded WPV. The overall NPENT and Sabin rates were 19.4% and 6.5% respectively. The highest and lowest temperature recorded for paired stool specimens with temperature excursion above 8°C were 36°C and 9°C, respectively. Of the 218 paired specimens with temperature excursion, 186(85.0%) were exposed for less than 24 hours, while 32(15.0%) were exposed for 24.0 to 40.3 hours. The highest temperature reading at which either a Sabin and/or NPENT was isolated from a stool specimen was 32.5°C and this excursion lasted for 40.3 hours. Overall, 50 (23%) yielded NPENT and 15 (6.9%) Sabin. Similarly, of the 824 paired specimens without temperature excursion, 152 (18.4%) yielded NPENT and 53 (6.4%) Sabin. Sabin-NPENT mixture and VDPV2 were isolated only from paired specimens without temperature excursion.

**Table 2:** laboratory yield of tracked paired stool specimens, reverse cold chain monitoring, Nigeria, November 2017 - December 2019

Variables	NPENT	Sabin	NPENT & Sabin	WPV	VDPV2	Negative	Total
Paired stool specimens with temperature excursion	50(23%)	15(6.9%)	0	0	0	153(70.1%)	218
Paired stool specimens without temperature excursion	150(18.2%)	51(6.2%)	2	0	3(0.4%)	618(75%)	824
**Total**	**200(19.2%)**	**66(6.3%)**	**2(0.2%)**	**0**	**3(0.3%)**	**771(74%)**	**1042**
Paired stool specimens with temperature excursion from non-insurgency affected states	8(8.5%)	3(3.2%)	0	0	0	83(88.3%)	94
Paired stool specimens without temperature excursion from non-insurgency affected states	60(17.5%)	17(5.0%)	2(0.6%)	0	0	263(76.9%)	342
**Total non-insurgency affected states**	**68(15.6%)**	**20(4.5%)**	**2(0.5%)**	**0**	**0**	**346(79.4%)**	**436**
Paired stool specimens with temperature excursion from insurgency affected states*	42(33.8%)	12(9.7%)	0	0	0	70(56.5%)	124
Paired stool specimens without temperature excursion from insurgency affected states*	90(18.7%)	34(7.1%)	0	0	3(0.6%)	355(73.6%)	482
**Total insurgency affected states**	**132(21.8%)**	**46(7.6%)**	**0**	**0**	**3(0.5%)**	**425(70.1%)**	**606**

* Insurgency affected states (Adamawa, Borno and Yobe) Footnote: Overall, enteroviruses were isolated from 65(29.8%) of 218 paired stool specimens with temperature excursion compared with 206(25%) of 824 paired stool specimens without temperature excursion (P=0.175)

Overall, enteroviruses were isolated from 65(29.8%) of 218 paired stool specimens with temperature excursion compared with 206(25%) of 824 paired stool specimens without temperature excursion (P=0.175) (Table 2). In insurgency affected states, the difference in enterovirus isolation between 54(43.5%) of 124 paired stool specimens with temperature excursion and 127(26.3%) of 482 without temperature excursion was statically significant (P= 0.0003). The difference in enterovirus isolation between 11(11.7%) of 94 paired stool specimens with temperature excursion and 79(23.1%) of 342 without excursion from non-insurgency affected states was also significant (P = 0.0229).

### Investigation of reverse cold chain with temperature excursion

A total of 163(75.0%) of the 218 tracked paired specimens with temperature excursion were investigated to identify the possible causes. The three main causes identified were icepack 84 (51.5%) and LogTag 40 (24.5%) related issues as well as caregivers tampering with the RCC, 35 (21.5%). Issues with the icepacks were due to delays in replacing icepacks, icepacks not well frozen, insufficient numbers of icepacks and failure to replace icepacks. Whereas, the LogTag related issues were due to faulty LogTags and unnecessary exposure of LogTags to ambient temperature during placement of stool specimens in specimen carrier and/or while replacing icepacks. In addition to the three main causes, other causes identified were delayed shipment to the laboratories 20(12.3%), knowledge gap of specimen couriers 13(8.0%) and opening of specimen carriers on transit by security officers 3(1.8%). These causes were not mutually exclusive.

## Discussion

This paper describes one of the several efforts to strengthen AFP surveillance and ensure that poliovirus transmission is not missed due to suboptimal maintenance of RCC for stool specimen storage and transport. The findings revealed that about 4 out of every 5 tracked paired stool specimens were maintained within the recommended temparature of +2 to +8°C from the point of collection to receipt at the polio laboratories. Almost all the specimens arrived in the laboratories within 72 hours of collection of second stool specimens. There was no significant difference in enterovirus isolation rates overall between paired stool specimens with and without temperature excursion.

Generally, findings from this study indicated that the DSNOs and ADSNOs maintained stool specimens within the recommended temperature range despite the enormous transportation and cold chain challenges, especially in insurgency affected states. Of particular interest, is that about 80% of paired stool specimens from AFP cases reported in insurgency affected states were maintained within recommended temperature from point of collection to arrival in the laboratories. Among other challenges, insurgency in northeast Nigeria has hugely affected immunization cold chain and AFP surveillance, as well as free movement of personnel, goods, and services within this area. Maintaining stool specimens within recommended temperatures against the backdrop of these challenges is, indeed, a reflection of the resilient nature of the Polio Eradication Initiative (PEI) program in Nigeria. Being the epicenter of poliovirus transmission in Nigeria from 2016, and the last sanctuary of WPV cases in the country, the insurgency affected areas attracted local and international concerns regarding the performance of AFP surveillance, the effectiveness of RCC in maintaining the viability of enteroviruses, and the likelihood of missing poliovirus transmission in these areas. In the context of these concerns, this finding is very important.

Since DSNOs and their assistants were responsible for collection and transportation of stool specimens to the laboratories, it was not possible to blind them to the temperature monitoring. Intuitively, it was likely for them to have made deliberate efforts to keep the specimens within recommended temperatures. From the programmatic perspective, this was highly desirable and corrective measures were implemented after investigating paired stool specimens with temperature excursion. A major public health implication of this is that RCC monitoring could be employed in settings with suboptimal RCC performance and possible low enterovirus isolation rate to spur improved performance.

Furthermore, our findings indicated that more than 99% of specimens arrived at polio laboratories within 72 hours of collection of the second stool specimens (target 90%), higher than what was reported by Adu et al. in Nigeria [12] and Odoom et al. [13] in Ghana. Transporting specimens from point of collection to the laboratories from these study areas within 72 hours is a herculean task, considering the vast and hard-to-reach nature of the country´s geographical landscape and security challenges. This might have partly contributed to the similarities in enterovirus detection rates in specimens with or without temperature excursions. Although, the DSNOs and ADSNOs that transported more than 99% of these stool specimens from the point of collection to the laboratories are supported by WHO to undertake this task, the resilience, dedication, commitment, and sacrifice of these officers in achieving this feat is commendable. In the light of the ongoing polio transition planning in Nigeria, the roles of these officers will be invaluable in strengthening integrated disease surveillance and response (IDSR) and other future disease elimination and/or eradication programmes if provided with the necessary resources.

We obtained NPENT rates of 23.0% and 18.0% for paired stool specimens without temperature excursion and with temperature excursion respectively, a difference that was not significant; both figures greater the NPENT operational target of 10% [3]. Similarly, we found no significant difference in the overall proportion of enterovirus isolated in specimens with and without temperature excursion. These findings demonstrate that although some specimens were exposed to temperatures higher than the recommended from point of collection to arrival at the laboratories, this exposure was not prolonged enough to render enteroviruses in stool specimens not viable [14]. The fact that almost all specimens arrived the laboratory within 72 hours further assures that it is unlikely for the laboratory to have missed any enterovirus including poliovirus in stool specimens.

In a study to determine viability of poliovirus exposed to different high temperature for varying time periods [14], the researchers found that WPV type 1 was highly stable at 25°C for 28 days and at 35°C for 16 days. Indeed, our result indicated that it was possible to isolate an enterovirus from stool specimens exposed to temperature of 32.5°C for up to 40.3 hours period which is very much shorter than the temperature described above for which polioviruses where viable. Considering the temperature range and the duration of exposure of stool specimens tracked in our intervention, it was unlikely for the laboratory to have missed enterovirus including poliovirus transmission in the study areas during the period of this intervention. Furthermore, all the LGAs in Adamawa, Borno and Yobe states were included in RCC monitoring because of the high probability of missing poliovirus transmission in these LGAs due to insurgency.

As expected, the enterovirus detection rate in specimens without temperature excursion in non-insurgency affected states was significantly higher. This was the opposite in insurgency affected LGAs. The unexpected significantly higher enterovirus rate in specimens with temperature excursion in insurgency affected LGAs can be explained by the physical and chemical characteristics of enteroviruses and difference in environment related conditions between insurgency affected LGAs and non-insurgency affected LGAs. Further studies maybe necessary to elucidate these observed differences.

Due to insecurity, only three-quarter of tracked paired stool specimens with temperature excursion were investigated. Our study found that in over half of the cases, the main reasons for temperature excursion were icepack related. However, LogTag related issues, as well as knowledge gap of specimen couriers also contributed to temperature excursions. In a study on RCC management in Nigeria, the authors highlighted provision of well frozen icepacks and re-educating the DSNOs on reverse cold chain management as key to ensuring optimal NPENT isolation [15]. Based on findings from investigation of specimen with temperature excursion, specific corrective measures were instituted. Some of these measures were capacity building for DSNOs and ADSNOs, ensuring availability of frozen icepacks, improved supportive supervision and education of caregivers on handling of specimen carriers. In addition, new specimen carriers (Giostyles) were provided to all 774 LGAs and 119 environmental surveillance sites.

We recognized some limitations in this intervention. Firstly, we used a non-probability sampling, as such our findings were not generalizable to the entire country but the study areas only. Secondly, since the DSNOs and their ADSNOs knew they were being monitored it is possible for them to have put in extra effort to ensure the RCC is maintained throughout the process. In this regard, observer bias might have played a role in influencing the enterovirus detection rate. Thirdly, we monitored paired stool specimens from 1,042(45%) of the 2,315 AFP detected from the selected LGAs during the intervention period. Only one LogTag was assigned to each LGA for this intervention, thus only one paired stool specimen could be tracked even in situations where multiple AFP cases were being investigated at the same time in a particular LGA. In addition, lost and damaged LogTags were reported in some LGAs. In such LGAs, although AFP investigation continued, stool specimens from AFP cases were not tracked until the LogTags were replaced. The increase in lost and damaged LogTags explains the decreased number of tracked specimens in 2019 compared to 2018. Lastly, we investigated three-quarters of paired stool specimens with temperature excursion. We were limited by insecurity due to insurgency; reasons for temperature excursions may be different in these areas.

## Conclusion

The maintenance of RCC for stool specimen storage and transport of the AFP surveillance system in the study area was optimal and effective in maintaining the viability of enterovirus including polioviruses in this area. It was therefore unlikely that poliovirus transmission was missed in the study area during the intervention period. Critical investment in the RCC capacity, building the capacity of surveillance officers periodically on maintaining adequate RCC, routine supportive supervision and ensuring specimens arrive the laboratories within 72 hours of collection of the second specimen, will maintain, further strengthen, and build confidence in the sensitivity of the AFP surveillance system in Nigeria.

### What is known about this topic

- A key component of the AFP surveillance is the reverse cold chain which is a system of storing and transporting stool specimens at the recommended temperature range from the point of collection to arrival at a WHO-accredited laboratory;
- Storing and transporting stool specimens in reverse cold chain outside the recommended temperature range could affect the viability of enteroviruses and potentially lower the likelihood of detecting enteroviruses in the laboratory.

### What this study adds

- Enteroviruses including poliovirus could be isolated from stool specimens exposed to temperature excursion from point of collection to the laboratory, especially if specimens arrive in the laboratory within 72 hours of collection of the second stool;
- The RCC component of the AFP surveillance system in the study area worked optimally with very low probability of undetected poliovirus transmission, in study area from 2017 to 2019.
